# Numerical Evaluation of the Effect of Geometric Tolerances on the High-Frequency Performance of Graphene Field-Effect Transistors

**DOI:** 10.3390/nano11113121

**Published:** 2021-11-19

**Authors:** Monica La Mura, Patrizia Lamberti, Vincenzo Tucci

**Affiliations:** Department of Information and Electrical Engineering and Applied Mathematics, University of Salerno, Via Giovanni Paolo II, 132, 84084 Fisciano, SA, Italy; plamberti@unisa.it (P.L.); vtucci@unisa.it (V.T.)

**Keywords:** design of experiments, GFET, graphene, high-frequency, RF devices, tolerance analysis

## Abstract

The interest in graphene-based electronics is due to graphene’s great carrier mobility, atomic thickness, resistance to radiation, and tolerance to extreme temperatures. These characteristics enable the development of extremely miniaturized high-performing electronic devices for next-generation radiofrequency (RF) communication systems. The main building block of graphene-based electronics is the graphene-field effect transistor (GFET). An important issue hindering the diffusion of GFET-based circuits on a commercial level is the repeatability of the fabrication process, which affects the uncertainty of both the device geometry and the graphene quality. Concerning the GFET geometrical parameters, it is well known that the channel length is the main factor that determines the high-frequency limitations of a field-effect transistor, and is therefore the parameter that should be better controlled during the fabrication. Nevertheless, other parameters are affected by a fabrication-related tolerance; to understand to which extent an increase of the accuracy of the GFET layout patterning process steps can improve the performance uniformity, their impact on the GFET performance variability should be considered and compared to that of the channel length. In this work, we assess the impact of the fabrication-related tolerances of GFET-base amplifier geometrical parameters on the RF performance, in terms of the amplifier transit frequency and maximum oscillation frequency, by using a design-of-experiments approach.

## 1. Introduction

The research in high-frequency electronics has been historically driven by the development of advanced radiofrequency (RF) wireless telecommunication systems.

Despite the advances in CMOS-based RF devices, unsolved issues related to losses and noise have determined the rise of III-V compound semiconductors technology, which made great achievements in high-frequency applications thanks to high electron mobility [1,2,3,4]. Meanwhile, graphene has already proven to have remarkable electron mobility and thermal conductivity, and the issues related to its zero-bandgap (that prevents graphene-based devices from turning off completely) are of secondary importance in analogue RF electronics [5,6,7,8,9]. Hence, a great number of graphene field effect transistors (GFETs) [6,10] has been proposed, pursuing a clear current saturation [11,12,13] and improved voltage gain [8,14] targeting RF applications [15,16,17,18,19,20,21], and demonstrating the capabilities of graphene-based RF electronics. As of now, cut-off frequencies in the range of *f*_T_ = 100–300 GHz [16,22], and above [23] have been experimentally demonstrated for GFETs, in line with the best silicon-based FETs. The GFET maximum oscillation frequency, though, is strongly limited below 70 GHz [20,24] by the poor current saturation, the high graphene/metal contact resistance at the Gate terminal [25,26,27], and the unclean graphene transfer process. Exceptionally, values as high as *f*_MAX_ = 200 GHz [28] were measured, which continue to be lower than the values theoretically achievable with graphene-based devices.

Even though these results are not comparable to the best-performing III–V HEMTs, graphene RF devices are still considered appealing due to the possibility of taking advantage of the GFET current ambipolarity, which enables a strong reduction in the transistor count and favours additional miniaturization capabilities [29]. This feature is extremely interesting, for example, for the aerospace field, particularly because it is accompanied by graphene’s inherent tolerance to radiation [30,31,32]. For these reasons, several examples of graphene-based RF devices have been proposed in recent years, including antennas [33,34], transmitters and receivers [35,36,37], modulators and demodulators [38,39,40,41,42,43], shields [44], power and signal amplifiers [45,46,47,48], mixers [49,50,51], and oscillators [52,53,54]. Important milestones were recently reached towards the large-scale fabrication of graphene electronic devices [55] and their integration into traditional semiconductor fabrication lines [56]. On this basis, graphene can be considered very promising for the development of breakthrough RF electronics.

In this scenario, one important challenge to address is the reliability of fabricated devices. The uncontrollable variations related to the manufacturing process tolerances determine an unavoidable non-uniformity across the devices, both fabricated on different wafers and on the same wafer. This inter-wafer and intra-wafer variability of the characteristics of the fabricated devices affects the uniformity of the performance of the fabricated devices. The process-related variations of nanomaterial-based electronic devices can be gathered in two categories of factors: factors related to the layout definition, and factors related to the material properties, as stated in [57]. The first category includes the geometrical parameters defined by the lithography (for the lateral dimensions) or by the growth/deposition process (for the vertical dimensions). The second category includes the parameters expressing the graphene quality (i.e., mobility, doping caused by traps and impurities, defects), which are determined by the capability of the growth or transfer process to not degrade the material electrical properties. These two categories of factors are independent and can be treated separately. In this paper, we focus on the first category of parameters.

Extracting a mathematical relationship between the GFET parameters variability and the performance variability, e.g., in the form of a regression model, is useful to predict the uncertainty resulting from the wafer processing. To optimize the number of runs necessary to get accurate modelling of the performance variation, design of experiments (DoE) techniques can be used [58,59,60].

In this work, we perform a tolerance analysis of a GFET common-source amplifier, originally proposed in [45] as the first high-frequency voltage amplifier obtained by using large-area CVD-grown graphene. The device performance is assessed by means of circuit simulations, designed according to a full factorial design of experiments, and performed using a large-signal charge-based compact model of a GFET described and validated in [61]. The Advanced Design System^®^ (Keysight Technologies, Inc., Santa Rosa, CA, USA) simulation environment is used by varying channel width, *W*, the channel length, *L*, and the top oxide thickness, *t*_OX_, in order to investigate the impact of geometry variations caused by the fabrication of process-related tolerances. Following the study presented in [62], where we discussed the impact of tolerances on the amplifier’s transconductance, *g*_m_, and output conductance, *g*_ds_, the influence of the same variations is reported here on the high-frequency performance described in terms of *f*_T_ and *f*_MAX_.

## 2. GFET Simulation Design

### 2.1. Input Parameter Space

The geometrical parameters determine the device input capacitance, output capacitance, and trans-capacitance, which limit the high-frequency performance of a field-effect transistor. In particular, the capacitances depend on the channel width, *W*, the channel length, *L*, and the top gate oxide thickness, *t*_OX_. The unevenness of these parameters, thus, impairs the uniformity of the fabricated devices’ high-frequency capabilities. In [57], it was observed that FETs based on nanowires and nanotubes are more robust to process-related geometry variations as compared to bulk silicon-based MOS devices and FinFETs, from the point of view of the direct current and of the input capacitance; the impact of the same parameters on the drain-source current of a GFET was assessed in [63]. Concerning graphene-based devices, the range of variation that should be considered for the geometrical factors is very process-dependent. The channel area is affected by an uncertainty generated either by the graphene sheet irregular shape (in the case of mechanical exfoliation and transfer of graphene flakes) [64], or by the lithography and/or etching steps (in the case of large-area CVD-grown graphene transfer) [65]. The accuracy of the thickness of the top-gate oxide depends on the thickness control capabilities of the growth or deposition technique and on the resulting roughness, and is also affected by inherent process variations [66]. 

In this work, the factors chosen for the tolerance analysis are *W*, *L*, and *t*_OX_, and in the absence of an initial estimate of the process tolerances, a variation ±Δ within the 10% of the nominal value is considered for each factor, in analogy with the approach proposed in [62,63,67,68,69].

The response variables of interest were computed in correspondence of all the combinations of the minimum value, centre value, and maximum value of each input factor, following a 3-factors, 3-levels full-factorial design of simulations. Hence, 3^3^ = 27 combinations of the input settings were considered. This approach allows accounting for simultaneous variations of all the considered input factors, enabling the investigation of possible interaction effects between the factors. In the proposed analysis, the factors are represented in the form of coded variables *x_i,c_*, where the minimum, nominal, and maximum values are represented by the values −1, 0, and 1, to provide an immediate matching with the regression model coefficients [60]. Including the centre point allows assessing the linearity of the response variable, with the scope of selecting the most suitable order for the regression model.

Table 1 reports the minimum, nominal, and maximum values of the simulation input parameters. The centre values for the three factors *W*, *L*, *t*_OX_ refer to the nominal design of the device described in [45] and investigated in [62,70].

### 2.2. Output Regression Model

The chosen performance indicators, computed in correspondence of the *n_c_* combinations of the input factors, are processed in accordance with the design of experiments techniques to evaluate the regression model coefficients. Depending on the linearity of the response variation with respect to the *m* = 3 factors *x_i,c_*, the regression model for the performance *y* obtainable from the 3-by-3 full factorial plan of simulation can be [60]:

- A first-order model, including only the linear dependence on the factors (main effects model):

*y* ≈ *y*_0_ + *β*_1_
*x*_1_ + *β*_2_
*x*_2_ + *β*_3_
*x*_3_ (1)

- A first-order model with interactions, including a small curvature in the response by means of the mixed product terms:

*y* ≈ *y*_0_ + *β*_1_
*x*_1_ + *β*_2_
*x*_2_ + *β*_3_
*x*_3_ + *β*_12_
*x*_1_
*x*_2_ + *β*_23_
*x*_2_
*x*_3_ + *β*_31_
*x*_3_
*x*_1_ (2)

- A second order model, including quadratic terms (response surface model):

*y* ≈ *y*_0_ + *β*_1_
*x*_1_ + *β*_2_
*x*_2_ + *β*_3_ x_3_ + *β*_11_
*x*_1_^2^ + *β*_22_
*x*_2_^2^ + *β*_33_
*x*_3_^2^ + *β*_12_
*x*_1_
*x*_2_ + *β*_23_
*x*_2_
*x*_3_ + *β*_31_
*x*_3_
*x*_1_ (3)

### 2.3. Response Variables

To assess the high-frequency operation capabilities of RF devices, the most common figures of merit are the transition frequency, *f*_T_, and the maximum oscillation frequency, *f*_MAX_.

In particular, *f*_T_ is defined as the frequency at which the current gain with the output in the short circuit condition reaches unity. By representing the common-source amplifier with a two-port network in which the input port is the gate-source terminal pair and the output port is the drain-source pair, the short-circuit current gain is the *h*_21_ parameter, which can be computed from the scattering parameters (*S*-parameters) matrix according to [71]:

*h*_21_ = −2*S*_21_ [(1 − *S*_11_) (1 + *S*_22_) + *S*_12_
*S*_21_]^−1^ (4)

The computation of the *S*-parameters is preferred because their evaluation does not require short-circuiting or open circuiting the input and output ports. These conditions are never satisfied perfectly at very high frequencies.

Despite its common use, *f*_T_ is not the most important figure of merit [72] in RF electronics. Amplifiers are useful as long as they are able to deliver power to the load, rather than current, and for this reason, it is important to also evaluate the transistor’s *f*_MAX_. This parameter is the frequency at which the maximum available gain (MAG), the frequency-dependent maximum power that can be transferred to the load in the impedance matching condition, reaches unity. *f*_MAX_ is, thus, the frequency over which the transistor is not able to amplify the input power in any case. This frequency is also called the maximum oscillation frequency because it is the frequency at which the transistor can trigger and sustain stable oscillations in oscillator circuit design. *f*_MAX_ is usually lower than *f*_T_, and the most interesting frequency between the two depends on the application.

### 2.4. Simulation Environment Setup

To assess the impact of the fabrication-related tolerance affecting the geometrical parameters on a GFET-based amplifier RF performance, a GFET small-signal model [73,74] can be used to compute the quantities of interest according to [6,29,73]

(5) $$f_{T}=\frac{g_{m}}{2\pi \left\{\left(C_{\mathrm{gs}}+C_{\mathrm{gd}}\right)\left[1+g_{\mathrm{ds}}\left(R_{S}+R_{D}\right)\right]+C_{\mathrm{gd}}g_{m}\left(R_{S}+R_{D}\right)\right\}}$$

(6) $$f_{\mathrm{MAX}}=\frac{g_{m}}{4\pi \left(C_{\mathrm{gs}}+C_{\mathrm{gd}}\right){\left[g_{\mathrm{ds}}\left(r_{i}+R_{S}+R_{G}\right)+g_{m}R_{G}\frac{C_{\mathrm{gd}}}{C_{\mathrm{gs}}+C_{\mathrm{gd}}}\right]}^{1/2\;}}$$

where *C*_gs_ is the gate-source capacitances and *C*_gd_ is the drain-source capacitance, *R*_S_, *R*_D_, *R*_G_, are the source, drain, and gate resistances, and *r*_i_ = 1/(2 $g_{m}$) is the intrinsic resistance [75]. 

Nevertheless, compact models for the simulation of the GFET electrical behaviour in large-signal operations have been developed and made compatible with most circuit simulators [61,76,77,78]. In this work, we use the charge-based large-signal GFET compact model presented in [61] and written in the hardware description language Verilog-A. This model preserves charge conservation and considers non-reciprocal self-capacitances and transcapacitances, contrarily to the Meyer’s and Meyer-like models commonly used [61]. The simulated device is the GFET common-source amplifier, made of high-quality single-layer CVD-grown graphene transferred onto a silicon oxide substrate, with an ultrathin high-*k* dielectric gate oxide [79] and a 6-finger embedded gate, presented in [45] as the first high-frequency voltage amplifier obtained by using large-area graphene and already simulated in [62,70]. The compact model used for the circuit simulations requires setting the input parameters related to the geometry, to the oxide material properties, and to the graphene characteristics. The nominal settings were obtained by Pasadas et al. in [70] by fitting the experimental I–V curve reported in [45], and are listed here in Table 2.

Concerning the resistance at the transistor’s terminals, they are taken into account by adding external lumped resistors. In [70], the values indicated for the drain and source contact resistances *R*_D_ and *R*_S_ for the nominal design of the considered device are dependent on the channel width and equal to *R*_D_ = *R*_S_ = 435 Ω μm, whereas the gate resistance *R*_G_ is a fixed resistance *R*_G_ = 14 Ω. Nevertheless, the contact resistance is known to impact strongly on the high-frequency limits of the GFET [80]. Therefore, in the performed simulations, the drain and source resistances and the gate resistance are increased proportionally to the channel width and to the channel length, respectively, in order to include the effect of the geometry variation. On the contrary, the dependence of the contact resistance upon other parameters related to the channel transport properties at different field intensities are not addressed here, since these properties are not related to the geometrical parameters that are the focus of this paper. 

**Figure 1:**
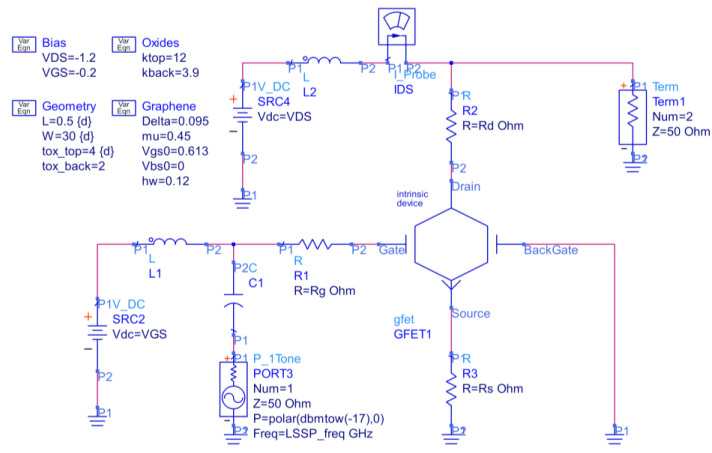
Schematic for the GFET amplifier large-signal *S*-parameters (LSSP) analysis.

The circuit schematic can be seen in Figure 1. 

**Figure 2:**
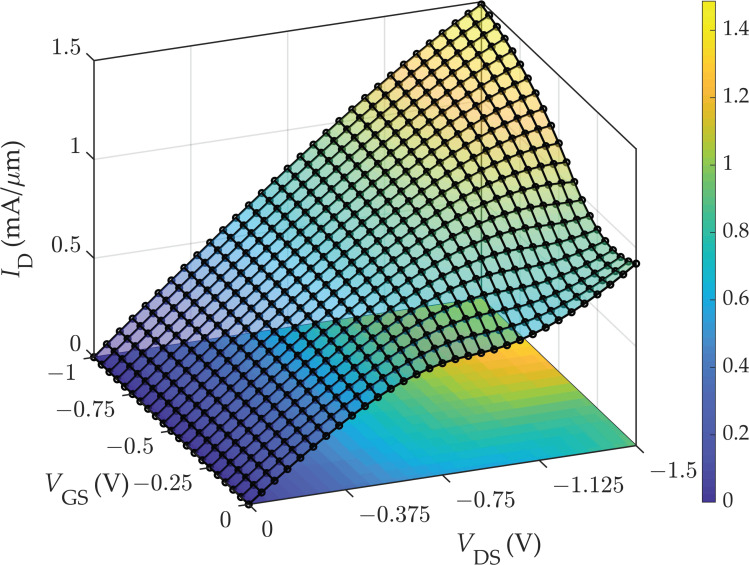
Surface plot of the drain current *I*_D_ computed by varying the *V*_GS_ and *V*_DS_.

**Figure 3:**
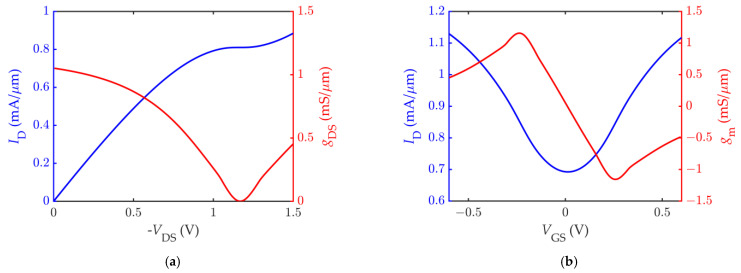
The DC characteristic of the simulated GFET: (**a**) the drain current *I*_D_ (in blue) against the drain-source voltage at *V*_GS_ = −0.2 V, with superimposed output conductance *g*_ds_ (in red); (**b**) the drain current *I*_D_ (in blue) against the gate-source voltage at *V*_DS_ = −1.2 V, with superimposed transconductance *g*_m_ (in red).

Simulations are run in the Advanced Design System—ADS (^®^Keysight, Inc., Santa Rosa, CA, USA) software environment, which performs a DC analysis to choose the bias point and large-signal *S*-parameters (LSSP) analysis to take into account the device nonlinearity in the computation of the *S*-parameters. Figure 2 shows the drain current *I*_D_ computed by varying the drain-source and gate-source bias voltage. As can be observed by viewing the surface curvature, the saturation of the drain current can be obtained in a certain bias region. Since the choice of the bias point is of great importance to achieve optimum performance [81], it was carefully chosen to achieve the maximum intrinsic voltage gain *A_V_* = *g*_m_ *g*_ds_^−1^. Searching for the optimal bias point, the applied *V*_DS_ was intentionally limited to prevent the effects of the carrier velocity saturation and the possible self-heating that intervene in high-field conditions, as these phenomena are not addressed by the model. On this basis, the bias point was set to *V*_GS_ = −0.2 V, *V*_DS_ = −1.2 V, as found in [62]. The output conductance *g*_ds_ on top of the drain current *I*_D_ output characteristic is shown in Figure 3a, and the transconductance *g*_m_ on top of the *I*_D_ transfer curve is shown in Figure 3b.

### 2.5. Validation of the Simulated GFET Behaviour

In order to validate the simulation results, the *f*_T_ and *f*_MAX_ obtained by the circuit simulator for the nominal design of the GFET were compared with the measured values reported in [45]. For this purpose, the analysis was performed by biasing the transistor at *V*_GS_ = −0.1 V, *V*_DS_ = −1.2 V, as reported in the paper. In addition, the values computed by means of the small-signal relations reported in Equations (5) and (6) are also reported. As can be observed, the results obtained by using Equations (5) and (6) agree neither with the experiment nor with the simulation, probably due to the nonlinear behaviour of the device and to the model being based on nonreciprocal capacitances. The simulation results replicate the measurements quite well, especially concerning the *f*_MAX_, as can be seen in Table 3. Differences between the simulation and the measurement can be caused by the imperfect value attributed to some of the graphene-related input parameters reported in Table 2, and can be reduced by applying optimization techniques to find the parameters’ values that improve the fitting of the measured current curves.

**Figure 4:**
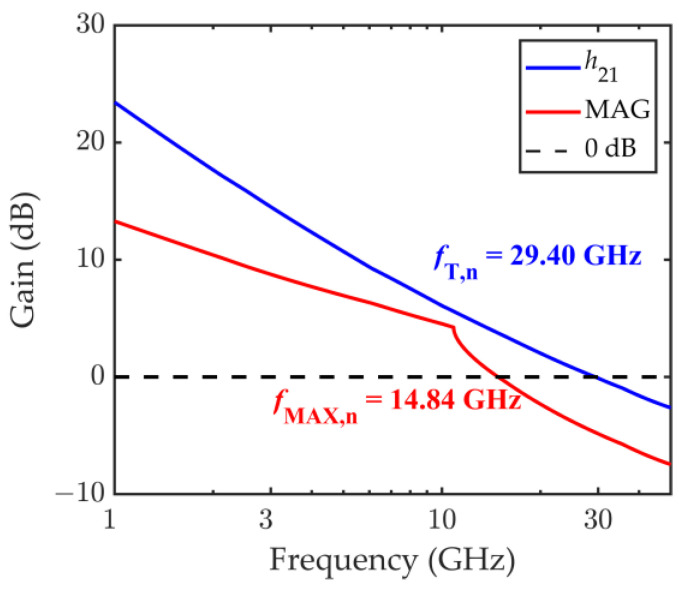
Short-circuit current gain *h*_21_ and maximum available gain MAG computed in correspondence of the nominal set of input parameters, at the bias point *V*_GS_ = −0.2 V, *V*_DS_ = −1.2 V. The nominal cut-off frequency is *f*_T,n_ = 29.40 GHz, and the nominal maximum oscillation frequency is *f*_MAX,n_ = 14.84 GHz.

The simulated *h*_21_ and MAG at the optimal bias point *V*_GS_ = −0.2 V, *V*_DS_ = −1.2 V, instead, return a nominal value for the *f*_T_ and *f*_MAX_ of *f*_T,n_ = 29.40 GHz and *f*_MAX,n_ = 14.84 GHz and are shown in Figure 4.

## 3. Tolerance Analysis Results

### 3.1. f_T_ Sensitivity

**Figure 5:**
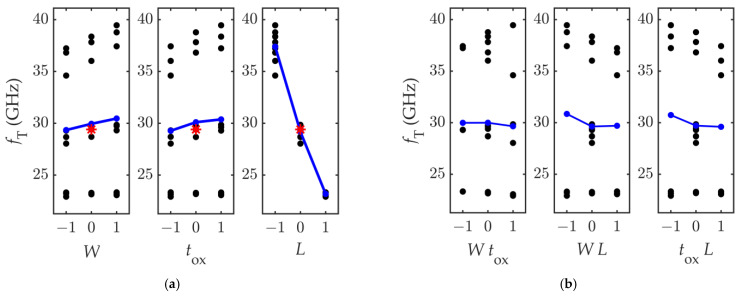
Computed values of *f*_T_ against (**a**) the input factors and (**b**) the factor-mixed products. The blue lines connect the *f*_T_ average values, and the red star marks the response computed at the nominal set of the input parameters.

To extract the *f*_T_ from the simulation results, the short-circuit current gain *h*_21_ was computed for the 27 combinations of the input factors, and the scattered data is plotted against the factors in Figure 5a, showing the main effects plot, and against the factor-mixed products in Figure 5b, showing the interaction effects plot. By looking at Figure 5 it can be concluded that the transition frequency *f*_T_ is by far more sensitive to the channel length *L* rather than to the other parameters, as the *L* factor variation causes the highest location shift of the mean performance, indicated by the blue dots for each level taken by the input factors. This result confirms expectations, since the peak cut-off frequency is reported to have a 1/*L* dependence in FETs with short gate lengths, and a 1/*L*^2^ dependence in FETs with long gate lengths [16]. The two other factors have the same influence on the *f*_T_, and both are much less effective than *L*. As can be observed from the *f*_T_ main effects and interaction effects values reported in Table 4, the main effect of the channel length *L*, ME_3_ = −7.11, is by far the highest contribution to the *f*_T_ variability. The interactions between the channel length *L* and the other two factors (i.e., IE_13_ and IE_23_) are very similar, and comparable to the main effects of *t*_OX_ and *W*, ME_1,_ and ME_2_. They are less than 10% of the main effect of the *L*, meaning that *W* and *t*_OX_ and their interactions with *L* impact the response variability by less than 10% of the impact of *L*.

Concerning the linearity of the response, the *f*_T_ variation induced by the variation of the factors of 10% is approximately linear; in fact, the blue line connecting the average *f*_T_ computed at the different levels of the input factors is pretty straight, and closely passes the nominal response *f*_T,n_.

To account for the slight nonlinearity of the response variable in the regression model, the interaction effects shown in Figure 5b can be considered. The interaction effects are computed by calculating the slope of the line connecting the average values of *f*_T_ computed when the product of the coded factors equals −1 and +1. The introduction of such effects can model the small curvatures in the response.

On this basis, the *f*_T_ variability can be modelled by:

*f*_T_ = 29.4 + 0.557 *W* + 0.549 *t*_OX_ − 7.11 *L* − 0.166 *W t*_OX_ − 0.575 *W L* − 0.57 *t*_OX_ *L* (7)

where *W*, *t*_OX,_ and *L* are varying between −1 and +1, following the coding reported in Table 1.

### 3.2. f_MAX_ Sensitivity

**Figure 6:**
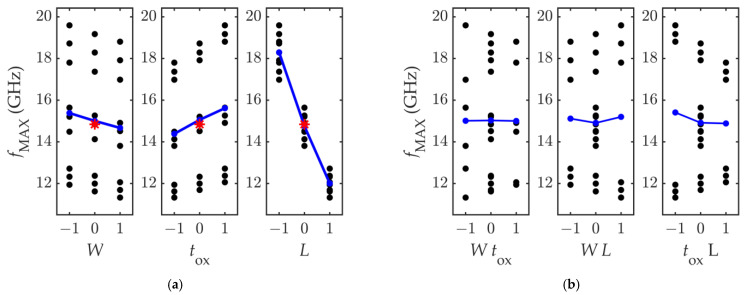
Computed values of *f_MAX_* against (**a**) the input factors and (**b**) the factor-mixed products. The blue lines connect the *f_MAX_* average values, and the red star marks the response computed at the nominal set of the input parameters.

The *f*_MAX_ variation in response to the variation of the input factors is shown in Figure 6a,b, which report the main effects plot and the interaction effect plot, respectively. As in the previous case, the blue lines connect the *f*_MAX_ average values computed in correspondence of each level of the factors, and the red star indicates the nominal response *f*_MAX,n_.

By looking at Figure 6, it can be observed that the factor most influential on the *f*_MAX_ is, as for the *f*_T_, the channel length *L*. However, contrarily to what was observed for *f*_T_, the increase of the channel width *W* causes a decrease of the *f*_MAX_. Another noticeable result is that, in this case, the response dependence on the three factors is very linear. In fact, the plots in Figure 6b show that there is no interaction between *W* and *t*_OX_, and that the interaction between *W* and *L* is one order of magnitude smaller than the lowest main effect. Moreover, while for the *f*_T_ the factors *W* and *t*_OX_ had a similar impact on the response, for the *f*_MAX_ it is observed that the *t*_OX_ is the second most influential parameter, as its main effect doubles the main effect of *W*, and is ≈20% the main effect of *L*. This is clearer by observing the computed values of the main effects and interaction effects reported in Table 5.

These values allow extracting the linear regression model representing the variability of the PF *f*_MAX_, which is:

*f*_MAX_ = 14.84 – 0.356 *W* + 0.615 *t*_OX_ − 3.14 *L* + 0.042 *W L* − 0.263 *t*_OX_ *L* (8)

## 4. Conclusions

An analysis of the impact on the fabrication-related tolerances of the GFET geometrical parameters was performed by means of designed circuit simulations.

The factor variation most influential on the transition frequency and the maximum oscillation frequency uniformity for a GFET-based common-source amplifier is the channel length *L*, coherently with the concept that the transistor high-frequency limit is inversely proportional to the time the carriers need to cross the channel. Reducing the channel length has great benefits on the transition frequency improvement and helps to improve the maximum frequency, too. Hence, being able to control the channel length reliably and applying all the possible measures to limit the occurrence of any uncontrollable phenomenon interfering with the channel length accuracy is the best way to reduce unwanted fluctuations of the fabricated transistors’ cut-off frequency, and therefore improve the intra-wafer and inter-wafer performance uniformity. The improvement provided by increasing the accuracy of the other geometrical parameters, instead, is very limited. In fact, this analysis has shown that the impact of the channel width *W* and the top oxide thickness *t*_OX_ on the *f_T_* is the same, and it is less than 10% of the impact of the channel length *L*. The interaction between *L* and the other two factors has an impact comparable to the *W* and *t*_OX_ main effect, and must therefore be included in the regression model for the *f_T_*. Concerning the *f*_MAX_, the *t*_OX_ is the second most influential factor, with the main effect that is about 20% of the *L* main effect. The *W* impacts on the *f*_MAX_ by less than 10% the impact of the *L*. A first-order regression model accounting for interaction between the factors is provided for both the considered performance indicators, allowing both the prediction of the expected variability when the tolerance of process parameters is known, and the definition of a region of acceptability for the factors’ tolerances when the variability of the observed performance is constrained.

In conclusion, the reduction of the variability of *W* and *t*_OX_ would improve the uniformity of the *f_T_* and *f*_MAX_ far less than a reduction of the variability of *L* by an equal percentage amount. The quantitative evaluation of this improvement can be done by using the provided mathematical relations between the quantities of interest. These considerations can support the cost/benefit analysis for the planning of investments to improve the ability of the manufacturing process to control the geometric parameters.

Further work includes the tolerance analysis of different GFET devices found in the literature, in order to compare the robustness of different device layouts and different processes to the fabrication-related tolerances. Moreover, the impact of graphene quality on the RF performance could be assessed quantitatively, providing the model with different inputs depending on the graphene quality indicators.

## Figures and Tables

**Table 1:** Input factors levels in the performed simulations.

Factor		Minimum	Nominal [45]	Maximum
*x*_1_	*W* (μm)	27	30	33
*x*_2_	*t*_OX_ (nm)	3.6	4	4.4
*x*_3_	*L* (μm)	0.45	0.5	0.55
Coded		−1	0	−1

**Table 2:** Input parameters of the circuit model at the nominal design point.

Parameter	Value [70]	Description
*L*	0.5 μm	Channel length
*W*	30 μm	Channel width
*t*_ox_	4 nm	Top oxide thickness
ε_top_	12	Top oxide relative permittivity
*V*_GS0_	0.613 V	Top gate voltage offset
Δ	0.095 eV	Electrostatic potential inhomogeneity due to electron-hole puddles
ℏω	0.12 eV	Effective energy of substrate optical phonon emission
μ	4500 cm^2^/Vs	Effective carrier mobility

**Table 3:** Simulated and measured *f*_T_, *f*_MAX_ at *V*_GS_ = −0.1 V, *V*_DS_ = −1.2 V.

	Simulated	Measured [45]	Computed
*f*_T_ (GHz)	9.3	8.2	7.2
*f*_MAX_ (GHz)	6.1	6.2	4.0

**Table 4:** *f*_T_ main effects and interaction effects.

	*x*_1_ (*W*)	*x*_2_ (*t*_OX_)	*x*_3_ (*L*)
*x*_1_ (*W*)	ME_1_ = 0.557	IE_12_ = −0.166	IE_13_ = −0.575
*x*_2_ (*t*_OX_)		ME_2_ = 0.549	IE_23_ = −0.570
*x*_3_ (*L*)			ME_3_ = −7.11

**Table 5:** *f*_MAX_ main effects and interaction effects.

	*x*_1_ (*W*)	*x*_2_ (*t*_OX_)	*x*_3_ (*L*)
***x*_1_(*W*)**	ME_1_ = 0.356	IE_12_ ≈ 0	IE_13_ = 0.042
***x*_2_(*t*_OX_)**		ME_2_ = 0.615	IE_23_ = −0.263
***x*_3_(*L*)**			ME_3_ = −3.14
